# Xenbase: Facilitating the Use of *Xenopus* to Model Human Disease

**DOI:** 10.3389/fphys.2019.00154

**Published:** 2019-02-26

**Authors:** Mardi J. Nenni, Malcolm E. Fisher, Christina James-Zorn, Troy J. Pells, Virgilio Ponferrada, Stanley Chu, Joshua D. Fortriede, Kevin A. Burns, Ying Wang, Vaneet S. Lotay, Dong Zhou Wang, Erik Segerdell, Praneet Chaturvedi, Kamran Karimi, Peter D. Vize, Aaron M. Zorn

**Affiliations:** ^1^Division of Developmental Biology, Cincinnati Children’s Hospital, Cincinnati, OH, United States; ^2^Department of Biological Sciences, University of Calgary, Calgary, AB, Canada; ^3^Institute of Ecology and Evolution, University of Oregon, Eugene, OR, United States

**Keywords:** *Xenopus*, Xenbase, model organism database, human disease, ontologies, oocyte, cell-free egg extract

## Abstract

At a fundamental level most genes, signaling pathways, biological functions and organ systems are highly conserved between man and all vertebrate species. Leveraging this conservation, researchers are increasingly using the experimental advantages of the amphibian *Xenopus* to model human disease. The online *Xenopus* resource, Xenbase, enables human disease modeling by curating the *Xenopus* literature published in PubMed and integrating these *Xenopus* data with orthologous human genes, anatomy, and more recently with links to the Online Mendelian Inheritance in Man resource (OMIM) and the Human Disease Ontology (DO). Here we review how Xenbase supports disease modeling and report on a meta-analysis of the published *Xenopus* research providing an overview of the different types of diseases being modeled in *Xenopus* and the variety of experimental approaches being used. Text mining of over 50,000 *Xenopus* research articles imported into Xenbase from PubMed identified approximately 1,000 putative disease- modeling articles. These articles were manually assessed and annotated with disease ontologies, which were then used to classify papers based on disease type. We found that *Xenopus* is being used to study a diverse array of disease with three main experimental approaches: cell-free egg extracts to study fundamental aspects of cellular and molecular biology, oocytes to study ion transport and channel physiology and embryo experiments focused on congenital diseases. We integrated these data into Xenbase Disease Pages to allow easy navigation to disease information on external databases. Results of this analysis will equip *Xenopus* researchers with a suite of experimental approaches available to model or dissect a pathological process. Ideally clinicians and basic researchers will use this information to foster collaborations necessary to interrogate the development and treatment of human diseases.

## *Xenopus* as a Model for Human Disease

*Xenopus* is used in biomedical research to study fundamental biological and pathological processes. The research community utilizes *Xenopus* to gain a deeper understanding of human disease through molecular analysis of disease-gene function and in-depth disease modeling. The advantages of the *Xenopus* model, including ease of housing, large oocyte and embryo size, high fecundity, rapid external development, and ease of genomic manipulation, make them invaluable tools to study the molecular basis of human development and disease. Compared to other aquatic models, this tetrapod is conservatively closer to humans with lungs, a three-chambered heart, and a close evolutionary relationship with mammals. *Xenopus* has been estimated to share 79% of the identified human disease genes (Hellsten et al., 2010; Khokha, 2012; Tandon et al., 2017). Compared to mammalian models, *Xenopus* is a rapid, cost-effective model with the ease of morpholino knock-down, the generation of efficient transgenics and targeted gene mutations using TALENs (transcription activator-like effector nucleases) or CRISPR/Cas (clustered regularly interspaced short palindromic repeats-CRISPR associated nucleases). Notably, many studies report the ease and efficiency of CRISPR/Cas modifications allowing phenotype analysis in the F0 generations of both *Xenopus laevis* and *Xenopus tropicalis* (Blitz et al., 2013; Bhattacharya et al., 2015; Wang et al., 2015). Similarly, CRISPR/Cas technology can be used to introduce small DNA fragments containing patient-specific variants for disease modeling in *Xenopus* (Aslan et al., 2017). In addition to in-depth disease modeling, these tools allow for efficient functional screening of genes identified in human genomic studies (Bhattacharya et al., 2015; Sater and Moody, 2017).

## Xenbase Support for Human Disease Modeling

Xenbase^1^ (RRID:SCR_003280), the *Xenopus* model organism database, is an NICHD-funded data repository with a major goal to help accelerate basic research and disease modeling (James-Zorn et al., 2018; Karimi et al., 2018). Xenbase collates all the *Xenopus* research data, and enhances the value of these data through high-quality curation. In this way Xenbase makes information, that would otherwise get buried in the scientific literature, computer searchable and highly integrated with an ever-growing knowledgebase. Xenbase links *Xenopus* genomic, epigenetic, mRNA and protein sequence with gene expression and gene function as well as physical reagents such as morpholinos and antibodies together with transgenic and mutant lines from the published literature. A second major goal of Xenbase is to enable the effective translation between *Xenopus* and human data by linking orthologous genes. In addition, Xenbase Gene Pages provide a link to the human ortholog gene-disease association via the Online Mendelian Inheritance in Man resource (OMIM^2^; RRID:SCR_006437), the comprehensive online catalog of genetically determined phenotypes. Additional links are made to inter-relate gene ontology (GO) (Ashburner et al., 2000; The Gene Ontology Consortium, 2017) and anatomy ontology terms.

In an ongoing effort to increase support for human disease modeling, Xenbase recently incorporated links to the Human Disease Ontology (DO^3^; RRID:SCR_000476), a standardized ontology for human disease terms and phenotype characteristics, with a long-term goal of merging disease annotations across species (Bello et al., 2018). DO integration facilitates annotation to a much broader scope of human diseases than OMIM alone, including non-Mendelian and environmentally induced diseases. Similarly, the hierarchical structure of the DO allows less specific high-level terms such as “cancer” in addition to more specific descendent terms such as “prostate cancer,” which can facilitate linking specific genes with classes of diseases.

**FIGURE 1:**
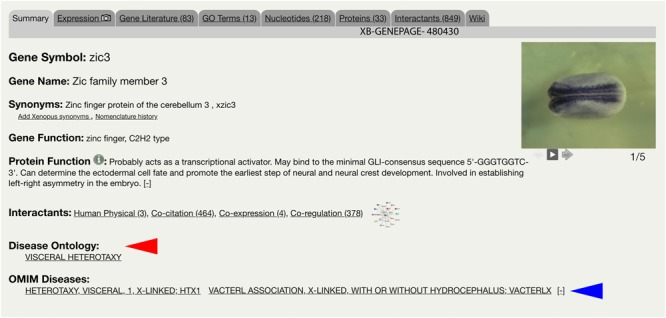
Xenbase Gene Page for *zic3*. Gene-disease annotations are located below Interactants on the Summary tab of the Xenbase Gene Page. Disease Ontology (DO) annotations (red arrowhead) are made via DO-OMIM cross reference or manual curation. OMIM annotations (blue arrowhead) are imported from the National Center for Biotechnology Information (NCBI). DO and OMIM terms link to Xenbase Disease Pages.

**FIGURE 2:**
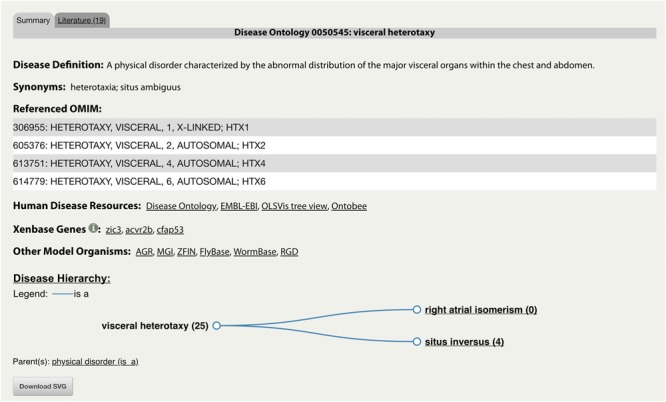
Xenbase Disease Page for “DOID:0050545: visceral heterotaxy.” An example of a new Disease Page with disease-specific supporting information including associated human and model organism resource links. The representative disease and its descendants are displayed in a Disease Hierarchy with the number of associated Xenbase articles in parentheses. The Literature tab provides a list of all associated Xenbase articles.

**FIGURE 3:**
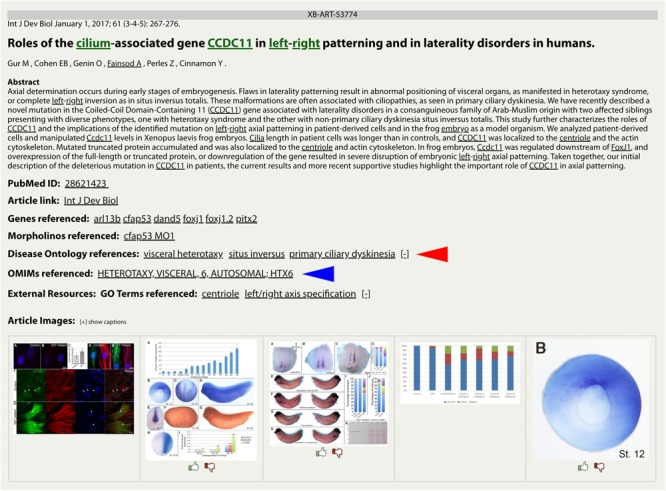
DO and OMIM references on a Xenbase Article Page. Disease terms link directly to the collated data on a Disease Page for DO (red arrowhead) annotations and OMIM (blue arrowhead) annotations. Multiple disease annotations can be seen by clicking the [+/–] toggle to show more or fewer results. Article Pages also list GO terms as keywords to cover the major topics of an article.

The integration of the DO into Xenbase provides new support to combine human disease information and *Xenopus* experimental data. The three main areas of integration are the Gene Page, Disease Page, and Article Page. *Xenopus* orthologs to human disease genes are given DO annotations on Gene Pages via DO-OMIM cross references and manual curation. For example, the *zic3* Gene Page contains the DO annotation for visceral heterotaxy (Figure 1). The “visceral heterotaxy” link leads to a new Xenbase feature, the Disease Page (Figure 2), where a user will find information including definitions, synonyms, and human disease resource links. Additional links to Xenbase genes and equivalent disease pages for other model organisms are provided. A compilation of DO annotation data from rat, mouse, zebrafish, fly, worm, and yeast is provided via the Alliance of Genome Resources (AGR) link^4^ (RRID:SCR_015850) (Howe et al., 2018). The Literature tab on the Disease Page provides a list of all disease-specific *Xenopus* literature. Similarly, Article Pages on Xenbase (Figure 3) contain links to any associated Disease Page. In addition to Disease Page links on Gene and Article Pages, diseases can be searched using the “Search Diseases” link on the Anatomy and Development tile of the homepage or via the search bar in the top right corner of the homepage. Type ahead will match terms or the ID number for a disease. A user can go directly to a specific Disease Page by highlighting the term or display all matches by searching a partial term.

## Text Mining and Annotation of *Xenopus*-Human Disease Literature

We used these newly implemented features on Xenbase to explore the landscape of human disease research that has used the *Xenopus* model system. Below we present a meta-analysis of approximately 1,000 human disease articles, from the corpus of 50,000+ articles in Xenbase, of which 554 were annotated with DO and/or OMIM terms, as well as gene, GO and anatomy assertions linking the *Xenopus* and human data. A meta-analysis of the resulting annotations was used to obtain an overview of the past and present use of *Xenopus* to study human disease. Finally, we discuss how these new features and the future integration of phenotypes will enhance support for researchers studying human disease.

A candidate human disease article list was established by three methods: (1) a PubMed search (*n* = 226), (2) by searching the full-text of articles imported into Xenbase (*n* = 466) using the text mining tool Textpresso v2.5^5^ (RRID:SCR_008737) (Muller et al., 2004) and (3) by selecting papers that had been identified from recent manual curation (*n* = 270). Details on the search parameters and keywords are provided in the Supplementary Material. The combined search identified 983 articles which were filtered for duplicates and then manually curated to a final set of 554 disease modeling papers based on one or more of the following conditions: (1) modeled human disease via gene knockdown, mutations or chemical manipulation in *Xenopus*; (2) examined the function of human disease-causing protein variants in *Xenopus*; (3) modeled a pathological process; (4) dissected the function of a gene or gene family implicated in human disease; (5) or pharmacological screen of compounds that cause or treat human disease.

## The Landscape of Human Disease Research in *Xenopus*

**FIGURE 4:**
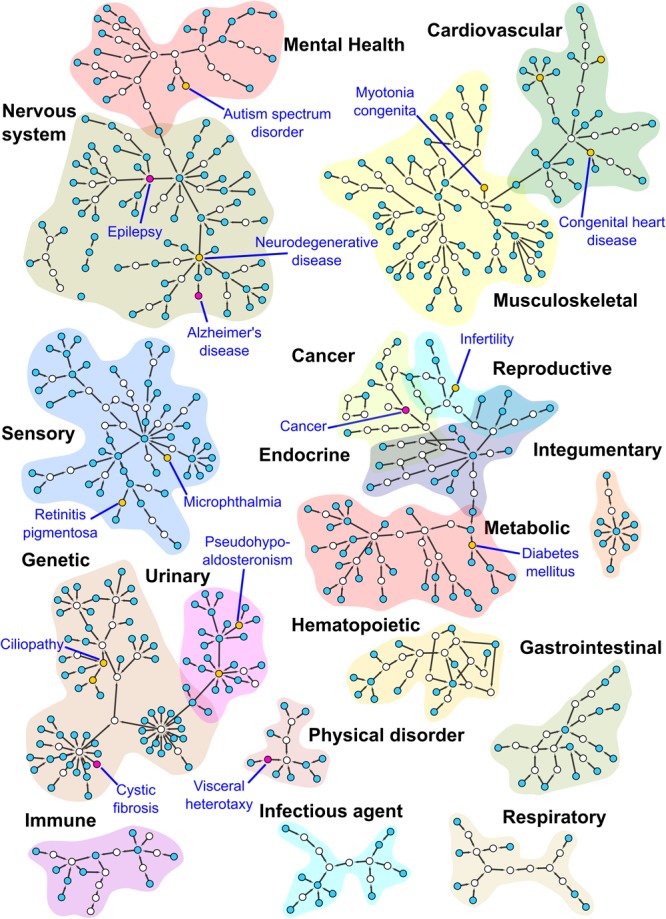
Subnetworks from the DO. This figure shows MCL clustered subnetworks from a subset of the DO, consisting of terms annotated during our curation of the Xenbase human disease corpus detailed in the Supplementary Material. Nodes in the network are colored according to the number of direct annotations to the term they represent. Empty nodes have no direct annotations, blue nodes 1–5, yellow nodes 6–14 and purple nodes 15 and higher. Purple nodes and the yellow node(s) with the highest number of annotations for each cluster have been labeled. Cluster regions corresponding to high level DO terms have been highlighted for contrast and labeled. Some small subnetworks and singleton nodes have been moved to proximity with the high level DO term to which they are associated.

Each of the 554 papers were curated with OMIM and DO terms and analyzed to obtain an overview of the major areas of human disease that were investigated using *Xenopus*. In total we annotated 887 DO terms from the 554 papers and then we assigned each of these DO annotations to one of 18 major disease groups using the hierarchical structure of the DO and selected the higher level DO terms (Table 1). A complete list of the DO terms with associated PubMed IDs is provided in Supplementary Table S1. We then clustered the DO terms using the Markov clustering (MCL) algorithm of clusterMaker2 (v1.2.1) and visualized the results with the network tool Cytoscape (Cytoscape; RRID:SCR_003032) (Shannon et al., 2003; Morris et al., 2011) (Figure 4 and Supplementary Material).

**Table 1:** High-level, less-specific, DO terms with summary numbers of total attributions, which includes direct annotations and indirect annotations from descendent, less-specific, terms and number of articles annotated.

DOID	DO term	Total Attributions	Articles
DOID:863	Nervous system disease	231	177
DOID:630	Genetic disease	125	101
DOID:0050155	Sensory system disease	80	63
DOID:17	Musculoskeletal system disease	74	61
DOID:1287	Cardiovascular system disease	70	62
DOID:162	Cancer	67	59
DOID:18	Urinary system disease	63	49
DOID:0014667	Disease of metabolism	44	36
DOID:150	Disease of mental health	41	36
DOID:0080015	Physical disorder	37	33
DOID:74	Hematopoietic system disease	28	14
DOID:77	Gastrointestinal system disease	24	19
DOID:28	Endocrine system disease	22	20
DOID:2914	Immune system disease	19	12
DOID:0050117	Disease by infectious agent	18	13
DOID:15	Reproductive system disease	13	10
DOID:16	Integumentary system disease	10	10
DOID:1579	Respiratory system disease	10	7

### Experimental Approaches for Disease Modeling in *Xenopus*

Our examination of the DO annotated literature revealed that *Xenopus* researchers tend to use three broad experimental approaches to study human disease: (1) the cell-free egg extract, (2) expression of proteins in oocytes, and (3) manipulations of developing *Xenopus* embryos and larva.

Cell-free egg extracts have long been used as a unique biochemical system to study fundamental components of the cell cycle including DNA check point, function of oncogenes, and tumor suppressor proteins (Cross and Powers, 2009; Willis et al., 2012; Hoogenboom et al., 2017). For example, researchers have used extracts to investigate biochemical mechanisms of genetic instability associated with variants in genes encoding DNA-damage checkpoint and repair proteins such as in Fanconi anemia (Sobeck et al., 2006; Stone et al., 2007; Landais et al., 2009; Sareen et al., 2012).

*Xenopus* oocytes, unfertilized eggs removed from the adult female, are used extensively to study a wide range of human diseases involved in ion transport and channel physiology, sometimes referred to as “channelopathies,” ranging from Alzheimer’s (Miledi et al., 2004; Ullah et al., 2015) to various forms of heart, kidney, and musculoskeletal disease (Felix, 2000; Vindas-Smith et al., 2016). Human proteins containing patient variants can be easily expressed in oocytes by micro-injection of recombinant mRNAs, and the large size (>1 mm) of the oocytes make them amenable to single-cell physiological analysis to determine how the variants affect protein function (Lehmann-Horn and Jurkat-Rott, 1999; Miledi et al., 2004; Sigel and Minier, 2005).

Finally, researchers use *Xenopus* embryos and tadpoles to model a broad range of human diseases from cardiovascular to mental health disorders. Experimental advantages include the large abundant externally developing embryos and transparent skin of later tadpoles, allowing one to examine organ development and disease, such as those occurring in the foregut, kidney, heart and brain (Salanga and Horb, 2015; Lienkamp, 2016; Dubey and Saint-Jeannet, 2017; Garfinkel and Khokha, 2017; Sater and Moody, 2017; Blum and Ott, 2018). Candidate human disease-causing genes are commonly studied by overexpression or knockdown of the orthologous *Xenopus* gene and analyzing the resulting phenotype, which if it resembles the human condition, can be used to study the details of pathogenesis or even provide a platform for therapeutics. Easy microinjection of *Xenopus* embryos enables gene knockdown by antisense morpholino oligos or more recently by CRISPR gene mutations (Bhattacharya et al., 2015; Shi et al., 2015; Wang et al., 2015), whereas wild type and mutant proteins can be overexpressed by mRNAs or tissue specific transgenics. Furthermore, the well-defined fate map (Moody, 1987a,b) allows researchers to perform targeted injection of specific tissues such as the nervous system or just the right side of the body. Many studies take advantage of unilateral injection, unique to *Xenopus*, to manipulate embryos and examine the phenotypic effects while the contralateral side functions as an internal control.

In addition to genetic mutations, the effects of environmental toxins are also studied in the embryo using established protocols such as the frog embryo teratogenesis assay in *Xenopus* (FETAX) (Dawson et al., 1989; Fort et al., 1989; Bantle et al., 1990; Morgan et al., 1996). In addition to the molecular and morphological analysis that the embryo and tadpole provide, behavioral studies are also employed to assess mental health function associated with human disease-causing toxins (Pratt and Khakhalin, 2013). A description of the predominant diseases studied and the associated *Xenopus* experimental approaches utilized are summarized below. A list of DO terms with associated *Xenopus* experimental approaches is provided in Supplementary Table S2.

### Nervous System Disease

The DO term “nervous system disease” had the greatest number of total attributions (*n* = 231). The specific nervous system diseases with the most DO annotations were epilepsy and Alzheimer’s disease. In general, two broad experimental approaches are used to study nervous system disease: (1) oocytes to study the function of mutant receptors and ion channels and (2) embryo manipulation to study neurodevelopmental disorders. For example, Simons et al. (2015) utilized the oocyte to characterize mutant or wildtype human KCNH1, a potassium channel implicated in Temple-Baraitser syndrome and epilepsy, and analyze the electrophysiological function by single-cell voltage-clamp. These experiments demonstrated that the variants lead to deleterious gain in function, which decreases the threshold of activation and delayed deactivation. Similarly, Miledi et al. (2004) demonstrated that cell membranes from post-mortem brains of Alzheimer’s patients can integrate into the *Xenopus* oocyte plasma membrane and maintain their neurotransmitter and voltage-gated channels, allowing researchers to investigate the cause and possible treatments for Alzheimer’s disease. Using embryo manipulations, Bell et al. (2011) utilized a developmental seizure model by treating embryos and tadpoles with the known convulsant, pentylenetetrazole (PTZ) to discover a novel neuroprotective role of polyamines in the developing brain.

### Genetic Disease

Genetic diseases, defined by the DO as a disease that has material basis in genetic variation in the human genome, had the second highest number of attributions (*n* = 125). Cystic fibrosis, an autosomal recessive disease, dominated the annotations in the genetic disease group with the majority of studies using the oocyte to examine ion transport and channel function. Cystic fibrosis is caused by variants in the *CFTR* gene, a chloride channel that regulates fluid flow across membranes that line vital organs such as lungs, intestine, and pancreas. Chen et al. (2012) used the oocyte to perform functional analyses of human single nucleotide polymorphisms (SNPs) in a potential CF modifier gene, *SLC26A9A*, encoding an anion transporter involved in chloride and bicarbonate exchange. These experiments shed light on the contribution of allelic variation in the pathophysiology of diseases affected by variants in *SLC26* family members, such as Pendred syndrome, an autosomal recessive disease characterized by hearing loss and euthyroid goiter. On the other hand, manipulation via knockdown and gain of function with morpholino and mRNA injections, respectively, were common techniques used to analyze genetic diseases such as branchiootorenal syndrome (BOS), an autosomal dominant disorder characterized by sensorineural, ear, branchial and renal defects. Knockdown of *pa2g4*, the *Xenopus* ortholog of a candidate human gene implicated in BOS, resulted in altered gene expression in neural crest and cranial placode providing insight into the molecular disease pathogenesis (Neilson et al., 2017). Similarly, Hoff et al. (2013) identified *ANKS6* variants in six families with nephronophthisis, an autosomal recessive cystic kidney disease that leads to adolescent renal failure, making this gene a strong disease-causing candidate. This was confirmed when targeted morpholino injections to knockdown *Xenopus anks6* in the intermediate mesoderm of one side of the embryos resulted in renal defects similar to patients. In general, screening for congenital disease-causing genes is a common use of *Xenopus* for many organ systems.

### Sensory System Disease

Sensory system diseases had the third highest number of DO attributions (*n* = 80), with the most common type being eye diseases. *Xenopus* embryos have long been used to study fundamental aspects of early eye development and are used to model diseases such as microphthalmia, retinitis pigmentosa, exudative vitreoretinopathy, and aniridia. Feehan et al. (2017) utilized CRISPR/Cas9-mediated mutations in *Xenopus* genes encoding rhodopsin to model both dominant and recessive forms of retinitis pigmentosa, a disease caused by retinal degeneration that leads to gradual loss of sight. Assays on retinal extracts and confocal microscopy were used to characterize the genotype-phenotype relationships. To a lesser extent, oocytes have also been used to study ion transport in eye diseases such as cataract. Staubli et al. (2017) utilized oocytes to assess the function of mutant versions of the human creatine transporter MCT12 by screening cDNAs from patients with age-related cataracts. A portion of the variants revealed decreased uptake of creatine, suggesting that non-invasive pharmacological interventions might be able to treat creatine deficiency in age related cataract.

### Musculoskeletal System Disease

The predominant diseases in the musculoskeletal category (*n* = 74) were myotonia congenita, a disease of chloride channels and congenital myasthenic syndrome, a neuromuscular junction disease, both of which used oocyte assays. Vindas-Smith et al. (2016) functionally characterized variants in the human gene, *CLCN1*, encoding a skeletal muscle chloride channel, found in non-syndromic myotonia congenita patients. Biophysical characterizations such as fast or slow gating, single channel conductance, current density and surface expression between wildtype and mutant channels expressed in *Xenopus* oocytes provided invaluable information on the complex genotype-phenotype relationship and provided molecular insight into potential therapeutics. Embryos have also been used to examine musculoskeletal diseases, such as Nager acrofacial dysostosis (Devotta et al., 2016) and idiopathic scoliosis (Lambert et al., 2009, 2013). Devotta et al. (2016) utilized antisense morpholinos to knockdown Sf3b4 function in *Xenopus* to generate an animal model of Nager acrofacial dysostosis (NAD), a disease characterized by underdeveloped cheek bones, very small lower jaw, cleft palate, defects in the middle ear, absent eyelashes, and a notch in the lower eyelid called a coloboma. The Sf3b4-depleted *Xenopus* embryos demonstrated reduced neural crest gene expression in the early embryo and resulted in hypoplastic neural crest-derived cartilages and craniofacial skeletal defects, similar to NAD patients. This has allowed researchers to dissect the pathogenesis of NAD and investigate targets of Sf3b4, one of the major genetic culprits of the disease.

### Cardiovascular System Disease

Congenital heart defect studies using embryos were the most frequently annotated DO term in the cardiovascular system disease category (*n* = 70). The transparent nature of *Xenopus* tadpole skin facilitates easy examination of heart development just 3 days post-fertilization (Duncan and Khokha, 2016). Mandel et al. (2010) utilized a cardiac-specific transgenic EGFP reporter to define the DNA cis-regulatory enhancers controlling expression of *tbx20*, the *Xenopus* ortholog of the human gene which has been linked to congenital heart disease. This allowed researchers to determine that the BMP/SMAD signaling pathway regulated *tbx20* expression in the heart and that disrupted BMP activity may also underlie other congenital heart defects. Importantly, the element was not specific to *Xenopus* and showed conservation in other species. The identification of this element provides researchers and clinicians with a non-coding region of *TBX20* in humans with the potential to contribute to congenital heart defects. Other cardiovascular diseases studied in *Xenopus* include long QT syndrome (LQTS), hypertension, and atrial fibrillation predominantly using oocytes to investigate the function of cardiac channel proteins. For example, Steffensen et al. (2015) functionally characterized human variants of unknown significance in two genes encoding potassium channels, *KCNQ1* and *KCNQ2*, from patients diagnosed with LQTS, an electrophysiological disorder of the heart that can lead to cardiac arrest or death. Single cell voltage clamping and confocal imaging of oocytes expressing these human proteins revealed loss-of-function phenotypes that resulted in abnormal electrophysiology as well as defects in cellular trafficking. These experiments highlighted the increased incidence of channel dysfunction in patients with LQTS.

### Cancer

All three *Xenopus* experimental approaches have been used to study cancer (*n* = 67). Each experimental approach offers unique advantages to study key cellular processes involved in tumorigenesis and metastasis such as division, differentiation, signaling and metabolism. The *Xenopus* embryo is a versatile model to characterize oncogenes and examine similarities between development and tumorigenesis (Hardwick and Philpott, 2015) with additional papers utilizing the cell-free egg extract (Cross and Powers, 2009) and oocyte (Nutt, 2012). A good example is Haynes-Gilmore et al. (2014) who used *Xenopus* embryos to study the tumor microenvironment by transplanting thymic lymphoid tumor cells under the dorsal skin of the tadpole. This xenograft model phenocopies many aspects of mammalian tumorigenesis and allows real time visualization of the tumor microenvironment including neovascularization, immune response, tissue rearrangements, and cellular migration. On the other hand, Joukov et al. (2006) utilized cell-free extracts to observe mitotic spindle assembly and reveal a previously unknown role for the heterodimeric tumor-suppressor BRCA1/BARD1 in this process, which is important for chromosome stability and tumor suppression. In general, hundreds of *Xenopus* studies have examined cell cycle and DNA-check point offering valuable information on general cancer mechanisms but did not pass our screening for specific human disease articles and are not represented here.

### Urinary System Disease

The *Xenopus* pronephric kidney offers a simplified model of the more complex mammalian kidney to study development, repair, and disease (Vize et al., 1997; Lienkamp, 2016). Top DO annotations in this category include kidney disease, nephrolithiasis, nephrogenic diabetes insipidus, pseudohypoaldosteronism, Liddle syndrome and Dent disease. The oocyte was the major experimental approach used to study urinary system diseases (*n* = 63) with a focus on defects in channel and ion transport that impact fluid homeostasis, blood filtration and urine production. For example, Ludwig et al. (2005) utilized the oocyte in structure-function studies of the human voltage-gated chloride channel, CIC-5, that when mutated, causes Dent disease, a renal tubular transport disease that leads to chronic kidney failure. Electrophysiology and imaging of oocytes expressing these channels provided insight into the importance of different amino acid sequences necessary for proper trafficking and recruitment throughout the trans-Golgi network.

### Diseases of Metabolism

Diabetes mellitus was the predominant condition annotated for diseases of metabolism (*n* = 44) with experiments utilizing either the embryos or oocytes. Pancreas development is highly conserved between *Xenopus* and mammals, making it an ideal model to study and screen genetic candidates involved in congenital pancreas defects (Kofent and Spagnoli, 2016) such as those occurring in type 1 diabetes mellitus. Simaite et al. (2014) took advantage of this high conservation with a combination of whole-genome sequencing with linkage analysis in a consanguineous family with early onset antibody-negative diabetes and morpholino knockdown of candidate orthologous genes in *Xenopus* to identify a variant in the patient gene *PCBD1* as the likely cause of pancreatic insufficiency and type 1 diabetes in these families. Oocytes have also been used to functionally characterize variants found in diabetes patients. For example, Proks et al. (2006) identified a variant in the *SUR1* gene, in a patient with DEND syndrome, which has a range of symptoms including neonatal diabetes. SUR1 is a regulatory subunit of a K(ATP) channel in pancreatic beta cells, and functional assays in oocytes revealed that the variants resulted in reduced channel sensitivity suggesting a novel genetic cause for neonatal diabetes. Other metabolic diseases studied in *Xenopus* oocytes included hemochromatosis, hypophosphatemia, and hypokalemic periodic paralysis.

### Disease of Mental Health

*Xenopus* embryos have long been used to study the effects of alcohol on development (Fainsod and Kot-Leibovich, 2018) and the resulting diseases of the mental health category (*n* = 41) including fetal alcohol spectrum disorder (FASD) or fetal alcohol syndrome (FAS). For example, Yelin et al. (2005) exposed embryos to various concentrations of alcohol at different time points to determine that the greatest period of sensitivity was early during gastrulation. At the molecular and morphological level, alcohol disrupted axial patterning and initial induction of the central nervous system. Further studies demonstrated the antagonistic effect of retinol (vitamin A) that resulted in phenotypic characteristics similar to those observed in humans with FAS including shortened rostro-caudal axis, microcephaly and microphthalmia.

*Xenopus* is also an ideal model to study mental health disorders resulting from defective nervous system development (Pratt and Khakhalin, 2013). Our analysis found six articles annotated as models for autism spectrum disorder (ASD). James et al. (2015) used a novel neurodegenerative model caused by valproic acid exposure during critical timepoints of neural circuit formation, which can lead to defective cognitive development, similar to those observed in ASD. Researchers took advantage of *Xenopus* behavioral assays known to be sensitive to abnormal circuit development, such as collision avoidance and schooling behavior, and found that valproic acid treatment resulted in tadpole behavioral abnormalities that were correlated with defects in brain morphology, dendritic structure and synaptic connectivity. These findings strengthened the hypothesis that changes in early neural circuitry can result in later behavioral deficits. Other studies have used oocytes to examine the impact of neurotransmitter function in ASD. Limon et al. (2008) examined the activity of GABA and glutamate neurotransmitter receptors from autistic brain tissue samples transplanted into *Xenopus* oocytes offering a novel approach to the study of autism, other neurological disorders, and drug discovery. Additional mental health disorders studied with *Xenopus* oocytes included schizophrenia, pain disorder, and intellectual disability.

### Physical Disorders

Many diseases in the physical disorder category (*n* = 37) involving defects in left-right patterning, neural tube closure or craniofacial birth defects have been studied in the frog embryo. Studies of left-right patterning disorders such as visceral heterotaxy and *situs inversus* predominated the physical disorder category. *Xenopus* studies have been instrumental in elucidating the molecular and cellular mechanisms of left-right patterning in the vertebrate embryo and have shown that this starts shortly after gastrulation with a group of mono-ciliated cells known as the left-right organizer (LRO) associated with the node or gastrocoel roof plate. Studies in *Xenopus* have defined the function of a candidate disease gene, *GALNT1*, identified in a patient with visceral heterotaxy (Boskovski et al., 2013). The quantitative live imaging of the LRO in *galnt1* knockdown embryos revealed disrupted cilia and defects in left-right asymmetry, suggesting a novel etiology for human heterotaxy. Similar to left-right patterning defects, *Xenopus* embryos have been used to study other physical disorders including craniofacial and neural tube defects, such as holoprosencephaly, microcephaly, and orofacial clefts because these structures can be easily observed in embryos several days after fertilization (Dickinson, 2016; Dubey and Saint-Jeannet, 2017).

### Hematopoietic System Disease

*Xenopus* was one of the early models for understanding developmental hematopoiesis (Dzierzak and Bigas, 2018) and our meta-analysis identified 28 total attributions to hematopoietic disease. Fanconi anemia had the greatest number of annotations, including four articles using cell-free egg extracts to study how the mechanisms of DNA-repair during replication and transcription are compromised in Fanconi anemia patients (Sobeck et al., 2006; Stone et al., 2007; Landais et al., 2009; Sareen et al., 2012). Similarly, Dominguez-Sola et al. (2007) took advantage of the nuclear-free egg extract system to define a non-transcriptional role of MYC in DNA replication which informed our mechanistic understanding of Burkitt lymphoma that is caused by chromosomal translocations involving MYC. Two other articles studied Diamond-Blackfan anemia with embryo experiments (Robson et al., 2016; Calo et al., 2018).

### Gastrointestinal System Disease

The predominant diseases of the gastrointestinal (GI) system studied in *Xenopus* (*n* = 24) were gastritis, cholestasis, and congenital secretory chloride diarrhea 1, all of which utilized the oocyte. Additional articles studying colorectal cancer, colon cancer and inflammatory bowel disease utilized either the oocyte or embryo studies. Notably, TALEN-mediated mutation of a *Xenopus* ortholog gene, *apc*, implicated in human colorectal cancer, was used to generate a *Xenopus* cancer model, enabling in-depth mechanistic analysis and therapeutic screenings which are not possible in other commonly used animal such as mice (Van Nieuwenhuysen et al., 2015).

### Endocrine System Disease

*Xenopus* has long been a model to study the role of endocrine hormones in metamorphosis, which are analogous to many of the hormonal changes occurring in the perinatal period of human birth (Yaoita et al., 1990; Buchholz, 2017). Endocrine hormones affect early embryonic organogenesis, brain development, metabolism and the reproductive system (Cossette and Drysdale, 2004; Bronchain et al., 2017). Moreover, endocrine disruption by chemical pollutants has been shown to lead to diseases of the reproductive system, thus a common focus for environmental toxicology studies using FETAX and other assays with *Xenopus* embryos (Mouche et al., 2017; Mughal et al., 2018). There were 22 DO attributions of endocrine system disease examining conditions such as Kallman syndrome, a disorder of sexual maturation, and hermaphroditism which encompasses disorders of sex chromosomes and sex determination, as well as the less specific term “endocrine system disease.”

### Diseases by Infectious Agent

The top DO terms annotated to diseases by infectious agent (*n* = 18) were related to malaria and HIV. These studies predominantly used *Xenopus* oocytes to study the effects of drugs. For example, Hertel et al. (2004) examined the mechanism of glucose transporter inhibition in oocytes, following HIV protease inhibitor treatment, a frequent side effect of these drugs that increases a patient’s risk for diabetes and cardiovascular disease.

### Immune, Integumentary, Respiratory, Reproductive System Diseases

Our meta-analysis of the *Xenopus* literature identified a diversity of other diseases that did not fall into major categories such as immune (*n* = 19), integumentary (*n* = 10), respiratory (*n* = 10), or reproductive (*n* = 13) systems. Notable examples include integumentary system diseases where Sangrithi et al. (2005) utilized cell-free egg extracts to link defective DNA replication with Rothmund-Thompson syndrome, a chromosome fragility disorder that is associated with skin disease. In the respiratory system disease category, Walentek et al. (2015) used morpholino to knockdown *atp4a*, a gastric ATPase that in humans is targeted by proton pump inhibitor (PPI) drugs to treat ulcers and other gastrointestinal diseases. The *Xenopus* studies helped explain why patients taking PPI have an increased risk of pneumonia. Knockdown of *Xenopus atp4a* resulted in multiciliated cell (MCC) defects of the *Xenopus* embryonic epidermis- a common model for mucociliary cells in the human airway epithelia (Walentek and Quigley, 2017), which are critical for ciliary-driven clearance of the lungs. These results suggest a possible causative linkage between PPIs and pneumonia due to defective function of the mucociliary epithelium of patient’s airways resulting in chronic congestion.

## Future Human Disease Support on Xenbase

**FIGURE 5:**
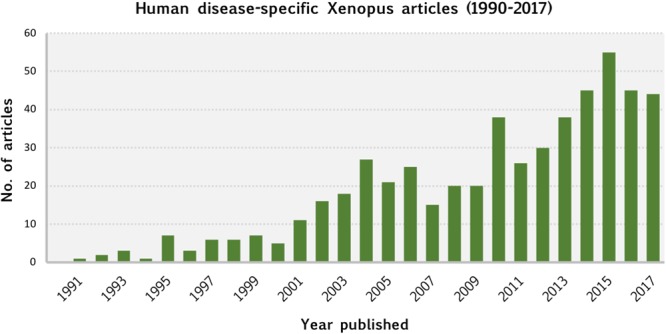
Human disease-specific *Xenopus* articles (1990–2017). This chart shows the number of articles published, by year, between 1990 and 2017 that our curation identified as utilizing *Xenopus* as a model system for studying human disease. Publication dates were obtained from NCBI’s PubMed database.

Our meta-analysis of the *Xenopus* literature was made possible by the curation and data integration of Xenbase. This comprehensive analysis revealed the breadth and depth of human disease modeling using *Xenopus* which continues to show an increasing trend (Figure 5). It is clear that *Xenopus* is an extremely versatile model system, and offers human disease researchers a broad suite of experimental approaches.

Modeling human disease is a major focus of the *Xenopus* research community and the recent incorporation of DO annotations into Xenbase facilitates this goal. Ongoing development in Xenbase includes the curation of *Xenopus* phenotypes using a new *Xenopus* Phenotype Ontology (XPO), which will enable us to directly link the results from *Xenopus* experiments to similar phenotypes in mice and men through cross references to the Mammalian Phenotype Ontology (MP) (Smith and Eppig, 2015) and the Human Phenotype Ontology (HPO) (Kohler et al., 2017), respectively. The XPO uses easy to understand anatomy-based (e.g., abnormal eye morphology) and GO term-based phrases (e.g., abnormal eye development) as well as clinical terms (e.g., microphthalmia). The planned strategy for future phenotype curation will align with other MODs with extensive manual curation of phenotypes such as the Mouse Genome Informatics (MGI) consortium^6^ (RRID:SCR_006460).

*Xenopus* high throughput datasets such as RNA-Seq and ChIP-Seq are increasing at a rapid pace. Xenbase is expanding support for these experimental approaches by processing all such public datasets from the Gene Expression Omnibus (GEO^7^; RRID:SCR_007303). Xenbase plans to integrate gene expression phenotypes with anatomical phenotypes. This gene expression as a phenotype (EaP) (Howe et al., 2017) approach will be used to describe experiments in which an experimental manipulation affects the expression of a gene in a tissue or embryo (e.g., *pax6* increased amount [in the] retina) as assayed by *in situ* hybridization or RNA-Seq assays. These new tools will enable researchers to use a systems biological approach to interrogate the gene regulatory networks underlying disease and therapeutic mechanisms, an area where the experimental advantages of the *Xenopus* system are well suited.

## Author Contributions

MN, MF, and CJ-Z conceptualized and designed the scope of the review, identified and curated the research articles, and analyzed and interpreted the results. MN drafted and wrote the manuscript. VP and MF generated the figures. MF, TP, and CJ-Z generated the data tables. MF, CJ-Z, KB, JF, AZ, and PV reviewed and revised the manuscript. AZ and PV, CO-PIs on the Xenbase grant, coordinated curation and development of the support for disease modeling, and assessed methodological quality. DW, TP, SC, VL, YW, and KK developed code and database support for disease pages and disease integration. All authors developed the support for disease modeling and approved the final manuscript as submitted.

## Conflict of Interest Statement

The authors declare that the research was conducted in the absence of any commercial or financial relationships that could be construed as a potential conflict of interest.

## Footnotes

1 http://www.xenbase.org

2 https://omim.org

3 http://www.disease-ontology.org

4 https://www.alliancegenome.org

5 http://textpresso.org

6 http://www.informatics.jax.org

7 https://www.ncbi.nlm.nih.gov/geo/

## References

[B1] Ashburner M. Ball C. A. Blake J. A. Botstein D. Butler H. Cherry J. M. (2000). Gene ontology: tool for the unification of biology. The Gene Ontology Consortium. *Nat. Genet.* 25 25–29. 10.1038/75556 10802651 PMC3037419

[B2] Aslan Y. Tadjuidje E. Zorn A. M. Cha S. W. (2017). High-efficiency non-mosaic CRISPR-mediated knock-in and indel mutation in F0 *Xenopus*. *Development* 144 2852–2858. 10.1242/dev.152967 28694259 PMC5560047

[B3] Bantle J. A. Fort D. J. Rayburn J. R. DeYoung D. J. Bush S. J. (1990). Further validation of FETAX: evaluation of the developmental toxicity of five known mammalian teratogens and non-teratogens. *Drug Chem. Toxicol.* 13 267–282. 10.3109/01480549009032286 1703942

[B4] Bell M. R. Belarde J. A. Johnson H. F. Aizenman C. D. (2011). A neuroprotective role for polyamines in a *Xenopus* tadpole model of epilepsy. *Nat. Neurosci.* 14 505–512. 10.1038/nn.2777 21378970

[B5] Bello S. M. Shimoyama M. Mitraka E. Laulederkind S. J. F. Smith C. L. Eppig J. T. (2018). Disease Ontology: improving and unifying disease annotations across species. *Dis. Model. Mech.* 11:dmm032839. 10.1242/dmm.032839 29590633 PMC5897730

[B6] Bhattacharya D. Marfo C. A. Li D. Lane M. Khokha M. K. (2015). CRISPR/Cas9: an inexpensive, efficient loss of function tool to screen human disease genes in *Xenopus*. *Dev. Biol.* 408 196–204. 10.1016/j.ydbio.2015.11.003 26546975 PMC4684459

[B7] Blitz I. L. Biesinger J. Xie X. Cho K. W. (2013). Biallelic genome modification in F(0) *Xenopus tropicalis* embryos using the CRISPR/Cas system. *Genesis* 51 827–834. 10.1002/dvg.22719 24123579 PMC4039559

[B8] Blum M. Ott T. (2018). *Xenopus*: an undervalued model organism to study and model human genetic disease. *Cells Tissues Organs* 10.1159/000490898 [Epub ahead of print]. 30092565

[B9] Boskovski M. T. Yuan S. Pedersen N. B. Goth C. K. Makova S. Clausen H. (2013). The heterotaxy gene GALNT11 glycosylates Notch to orchestrate cilia type and laterality. *Nature* 504 456–459. 10.1038/nature12723 24226769 PMC3869867

[B10] Bronchain O. J. Chesneau A. Monsoro-Burq A. H. Jolivet P. Paillard E. Scanlan T. S. (2017). Implication of thyroid hormone signaling in neural crest cells migration: evidence from thyroid hormone receptor beta knockdown and NH3 antagonist studies. *Mol. Cell. Endocrinol.* 439 233–246. 10.1016/j.mce.2016.09.007 27619407

[B11] Buchholz D. R. (2017). *Xenopus* metamorphosis as a model to study thyroid hormone receptor function during vertebrate developmental transitions. *Mol. Cell. Endocrinol.* 459 64–70. 10.1016/j.mce.2017.03.020 28363743

[B12] Calo E. Gu B. Bowen M. E. Aryan F. Zalc A. Liang J. (2018). Tissue-selective effects of nucleolar stress and rDNA damage in developmental disorders. *Nature* 554 112–117. 10.1038/nature25449 29364875 PMC5927778

[B13] Chen A. P. Chang M. H. Romero M. F. (2012). Functional analysis of nonsynonymous single nucleotide polymorphisms in human SLC26A9. *Hum. Mutat.* 33 1275–1284. 10.1002/humu.22107 22544634 PMC3399991

[B14] Cossette S. M. Drysdale T. A. (2004). Early expression of thyroid hormone receptor beta and retinoid X receptor gamma in the *Xenopus* embryo. *Differentiation* 72 239–249. 10.1111/j.1432-0436.2004.07205006.x 15270780

[B15] Cross M. K. Powers M. A. (2009). Learning about cancer from frogs: analysis of mitotic spindles in *Xenopus* egg extracts. *Dis. Model. Mech.* 2 541–547. 10.1242/dmm.002022 19892884 PMC2773725

[B16] Dawson D. A. Fort D. J. Newell D. L. Bantle J. A. (1989). Developmental toxicity testing with FETAX: evaluation of five compounds. *Drug Chem. Toxicol.* 12 67–75. 10.3109/01480548908999144 2714209

[B17] Devotta A. Juraver-Geslin H. Gonzalez J. A. Hong C. S. Saint-Jeannet J. P. (2016). Sf3b4-depleted *Xenopus* embryos: a model to study the pathogenesis of craniofacial defects in Nager syndrome. *Dev. Biol.* 415 371–382. 10.1016/j.ydbio.2016.02.010 26874011 PMC4914463

[B18] Dickinson A. J. (2016). Using frogs faces to dissect the mechanisms underlying human orofacial defects. *Semin. Cell Dev. Biol.* 51 54–63. 10.1016/j.semcdb.2016.01.016 26778163 PMC4798872

[B19] Dominguez-Sola D. Ying C. Y. Grandori C. Ruggiero L. Chen B. Li M. (2007). Non-transcriptional control of DNA replication by c-Myc. *Nature* 448 445–451. 10.1038/nature05953 17597761

[B20] Dubey A. Saint-Jeannet J. P. (2017). Modeling human craniofacial disorders in *Xenopus*. *Curr. Pathobiol. Rep.* 5 79–92. 10.1007/s40139-017-0128-8 28255527 PMC5327820

[B21] Duncan A. R. Khokha M. K. (2016). *Xenopus* as a model organism for birth defects-Congenital heart disease and heterotaxy. *Semin. Cell Dev. Biol.* 51 73–79. 10.1016/j.semcdb.2016.02.022 26910255 PMC4809202

[B22] Dzierzak E. Bigas A. (2018). Blood development: hematopoietic stem cell dependence and independence. *Cell Stem Cell* 22 639–651. 10.1016/j.stem.2018.04.015 29727679

[B23] Fainsod A. Kot-Leibovich H. (2018). *Xenopus* embryos to study fetal alcohol syndrome, a model for environmental teratogenesis. *Biochem. Cell Biol.* 96 77–87. 10.1139/bcb-2017-0219 29069552

[B24] Feehan J. M. Chiu C. N. Stanar P. Tam B. M. Ahmed S. N. Moritz O. L. (2017). Modeling dominant and recessive forms of retinitis pigmentosa by editing three *Rhodopsin*-encoding genes in *Xenopus laevis* using crispr/Cas9. *Sci. Rep.* 7:6920. 10.1038/s41598-017-07153-4 28761125 PMC5537283

[B25] Felix R. (2000). Channelopathies: ion channel defects linked to heritable clinical disorders. *J. Med. Genet.* 37 729–740. 10.1136/jmg.37.10.729 11015449 PMC1757150

[B26] Fort D. J. James B. L. Bantle J. A. (1989). Evaluation of the developmental toxicity of five compounds with the frog embryo teratogenesis assay: *Xenopus* (FETAX) and a metabolic activation system. *J. Appl. Toxicol.* 9 377–388. 10.1002/jat.2550090603 2613998

[B27] Garfinkel A. M. Khokha M. K. (2017). An interspecies heart-to-heart: using *Xenopus* to uncover the genetic basis of congenital heart disease. *Curr. Pathobiol. Rep.* 5 187–196. 10.1007/s40139-017-0142-x 29082114 PMC5658036

[B28] Hardwick L. J. Philpott A. (2015). An oncologists friend: how *Xenopus* contributes to cancer research. *Dev. Biol.* 408 180–187. 10.1016/j.ydbio.2015.02.003 25704511 PMC4684227

[B29] Haynes-Gilmore N. Banach M. Edholm E. S. Lord E. Robert J. (2014). A critical role of non-classical MHC in tumor immune evasion in the amphibian *Xenopus* model. *Carcinogenesis* 35 1807–1813. 10.1093/carcin/bgu100 24776220 PMC4123649

[B30] Hellsten U. Harland R. M. Gilchrist M. J. Hendrix D. Jurka J. Kapitonov V. (2010). The genome of the Western clawed frog *Xenopus tropicalis*. *Science* 328 633–636. 10.1126/science.1183670 20431018 PMC2994648

[B31] Hertel J. Struthers H. Horj C. B. Hruz P. W. (2004). A structural basis for the acute effects of HIV protease inhibitors on GLUT4 intrinsic activity. *J. Biol. Chem.* 279 55147–55152. 10.1074/jbc.M410826200 15496402 PMC1403823

[B32] Hoff S. Halbritter J. Epting D. Frank V. Nguyen T. M. van Reeuwijk J. (2013). ANKS6 is a central component of a nephronophthisis module linking NEK8 to INVS and NPHP3. *Nat. Genet.* 45 951–956. 10.1038/ng.2681 23793029 PMC3786259

[B33] Hoogenboom W. S. Klein Douwel D. Knipscheer P. (2017). *Xenopus* egg extract: a powerful tool to study genome maintenance mechanisms. *Dev. Biol.* 428 300–309. 10.1016/j.ydbio.2017.03.033 28427716

[B34] Howe D. G. Blake J. A. Bradford Y. M. Bult C. J. Calvi B. R. Engel S. R. (2018). Model organism data evolving in support of translational medicine. *Lab. Anim.* 47 277–289. 10.1038/s41684-018-0150-4 30224793 PMC6322546

[B35] Howe D. G. Bradford Y. M. Eagle A. Fashena D. Frazer K. Kalita P. (2017). The Zebrafish Model Organism Database: new support for human disease models, mutation details, gene expression phenotypes and searching. *Nucleic Acids Res.* 45 D758–D768. 10.1093/nar/gkw1116 27899582 PMC5210580

[B36] James E. J. Gu J. Ramirez-Vizcarrondo C. M. Hasan M. Truszkowski T. L. Tan Y. (2015). Valproate-induced neurodevelopmental deficits in *Xenopus laevis* tadpoles. *J. Neurosci.* 35 3218–3229. 10.1523/jneurosci.4050-14.2015 25698756 PMC4331635

[B37] James-Zorn C. Ponferrada V. Fisher M. E. Burns K. Fortriede J. Segerdell E. (2018). Navigating xenbase: an integrated *Xenopus* genomics and gene expression database. *Methods Mol. Biol.* 1757 251–305. 10.1007/978-1-4939-7737-6_10 29761462 PMC6853059

[B38] Joukov V. Groen A. C. Prokhorova T. Gerson R. White E. Rodriguez A. (2006). The BRCA1/BARD1 heterodimer modulates ran-dependent mitotic spindle assembly. *Cell* 127 539–552. 10.1016/j.cell.2006.08.053 17081976

[B39] Karimi K. Fortriede J. D. Lotay V. S. Burns K. A. Wang D. Z. Fisher M. E. (2018). Xenbase: a genomic, epigenomic and transcriptomic model organism database. *Nucleic Acids Res.* 46 D861–D868. 10.1093/nar/gkx936 29059324 PMC5753396

[B40] Khokha M. K. (2012). *Xenopus* white papers and resources: folding functional genomics and genetics into the frog. *Genesis* 50 133–142. 10.1002/dvg.22015 22287484

[B41] Kofent J. Spagnoli F. M. (2016). *Xenopus* as a model system for studying pancreatic development and diabetes. *Semin. Cell Dev. Biol.* 51 106–116. 10.1016/j.semcdb.2016.01.005 26806634

[B42] Kohler S. Vasilevsky N. A. Engelstad M. Foster E. McMurry J. Ayme S. (2017). The human phenotype ontology in 2017. *Nucleic Acids Res.* 45 D865–D876. 10.1093/nar/gkw1039 27899602 PMC5210535

[B43] Lambert F. M. Malinvaud D. Glaunes J. Bergot C. Straka H. Vidal P. P. (2009). Vestibular asymmetry as the cause of idiopathic scoliosis: a possible answer from *Xenopus*. *J. Neurosci.* 29 12477–12483. 10.1523/jneurosci.2583-09.2009 19812323 PMC6665096

[B44] Lambert F. M. Malinvaud D. Gratacap M. Straka H. Vidal P. P. (2013). Restricted neural plasticity in vestibulospinal pathways after unilateral labyrinthectomy as the origin for scoliotic deformations. *J. Neurosci.* 33 6845–6856. 10.1523/jneurosci.4842-12.2013 23595743 PMC6618882

[B45] Landais I. Hiddingh S. McCarroll M. Yang C. Sun A. Turker M. S. (2009). Monoketone analogs of curcumin, a new class of Fanconi anemia pathway inhibitors. *Mol. Cancer* 8:133. 10.1186/1476-4598-8-133 20043851 PMC2807854

[B46] Lehmann-Horn F. Jurkat-Rott K. (1999). Voltage-gated ion channels and hereditary disease. *Physiol. Rev.* 79 1317–1372. 10.1152/physrev.1999.79.4.1317 10508236

[B47] Lienkamp S. S. (2016). Using *Xenopus* to study genetic kidney diseases. *Semin. Cell Dev. Biol.* 51 117–124. 10.1016/j.semcdb.2016.02.002 26851624

[B48] Limon A. Reyes-Ruiz J. M. Miledi R. (2008). Microtransplantation of neurotransmitter receptors from postmortem autistic brains to *Xenopus* oocytes. *Proc. Natl. Acad. Sci. U.S.A.* 105 10973–10977. 10.1073/pnas.0804386105 18645182 PMC2504813

[B49] Ludwig M. Doroszewicz J. Seyberth H. W. Bokenkamp A. Balluch B. Nuutinen M. (2005). Functional evaluation of Dent’s disease-causing mutations: implications for ClC-5 channel trafficking and internalization. *Hum. Genet.* 117 228–237. 10.1007/s00439-005-1303-2 15895257

[B50] Mandel E. M. Kaltenbrun E. Callis T. E. Zeng X. X. Marques S. R. Yelon D. (2010). The BMP pathway acts to directly regulate Tbx20 in the developing heart. *Development* 137 1919–1929. 10.1242/dev.043588 20460370 PMC2867324

[B51] Miledi R. Duenas Z. Martinez-Torres A. Kawas C. H. Eusebi F. (2004). Microtransplantation of functional receptors and channels from the Alzheimer’s brain to frog oocytes. *Proc. Natl. Acad. Sci. U.S.A.* 101 1760–1763. 10.1073/pnas.0308224100 14749517 PMC341849

[B52] Moody S. A. (1987a). Fates of the blastomeres of the 16-cell stage *Xenopus* embryo. *Dev. Biol.* 119 560–578. 10.1016/0012-1606(87)90059-5 3803718

[B53] Moody S. A. (1987b). Fates of the blastomeres of the 32-cell-stage *Xenopus* embryo. *Dev. Biol.* 122 300–319. 3596014 10.1016/0012-1606(87)90296-x

[B54] Morgan M. K. Scheuerman P. R. Bishop C. S. Pyles R. A. (1996). Teratogenic potential of atrazine and 2,4-D using FETAX. *J. Toxicol. Environ. Health* 48 151–168. 10.1080/009841096161401 8642623

[B55] Morris J. H. Apeltsin L. Newman A. M. Baumbach J. Wittkop T. Su G. (2011). *clusterMaker*: a multi-algorithm clustering plugin for Cytoscape. *BMC Bioinformatics* 12:436. 10.1186/1471-2105-12-436 22070249 PMC3262844

[B56] Mouche I. Malesic L. Gillardeaux O. (2017). FETAX assay for evaluation of developmental toxicity. *Methods Mol. Biol.* 1641 311–324. 10.1007/978-1-4939-7172-5_17 28748472

[B57] Mughal B. B. Demeneix B. A. Fini J. B. (2018). Evaluating thyroid disrupting chemicals in vivo using *Xenopus laevis*. *Methods Mol. Biol.* 1801 183–192. 10.1007/978-1-4939-7902-8_15 29892825

[B58] Muller H. M. Kenny E. E. Sternberg P. W. (2004). Textpresso: an ontology-based information retrieval and extraction system for biological literature. *PLoS Biol.* 2:e309. 10.1371/journal.pbio.0020309 15383839 PMC517822

[B59] Neilson K. M. Abbruzzesse G. Kenyon K. Bartolo V. Krohn P. Alfandari D. (2017). Pa2G4 is a novel Six1 co-factor that is required for neural crest and otic development. *Dev. Biol.* 421 171–182. 10.1016/j.ydbio.2016.11.021 27940157 PMC5221411

[B60] Nutt L. K. (2012). The *Xenopus* oocyte: a model for studying the metabolic regulation of cancer cell death. *Semin. Cell Dev. Biol.* 23 412–418. 10.1016/j.semcdb.2012.03.015 22507445

[B61] Pratt K. G. Khakhalin A. S. (2013). Modeling human neurodevelopmental disorders in the *Xenopus* tadpole: from mechanisms to therapeutic targets. *Dis. Model. Mech.* 6 1057–1065. 10.1242/dmm.012138 23929939 PMC3759326

[B62] Proks P. Arnold A. L. Bruining J. Girard C. Flanagan S. E. Larkin B. (2006). A heterozygous activating mutation in the sulphonylurea receptor SUR1 (ABCC8) causes neonatal diabetes. *Hum. Mol. Genet.* 15 1793–1800. 10.1093/hmg/ddl101 16613899

[B63] Robson A. Owens N. D. Baserga S. J. Khokha M. K. Griffin J. N. (2016). Expression of ribosomopathy genes during *Xenopus tropicalis* embryogenesis. *BMC Dev. Biol.* 16:38. 10.1186/s12861-016-0138-5 27784267 PMC5081970

[B64] Salanga M. C. Horb M. E. (2015). *Xenopus* as a model for GI/pancreas disease. *Curr. Pathobiol. Rep.* 3 137–145. 10.1007/s40139-015-0076-0 26236566 PMC4518857

[B65] Sangrithi M. N. Bernal J. A. Madine M. Philpott A. Lee J. Dunphy W. G. (2005). Initiation of DNA replication requires the RECQL4 protein mutated in Rothmund-Thomson syndrome. *Cell* 121 887–898. 10.1016/j.cell.2005.05.015 15960976

[B66] Sareen A. Chaudhury I. Adams N. Sobeck A. (2012). Fanconi anemia proteins FANCD2 and FANCI exhibit different DNA damage responses during S-phase. *Nucleic Acids Res.* 40 8425–8439. 10.1093/nar/gks638 22753026 PMC3458572

[B67] Sater A. K. Moody S. A. (2017). Using *Xenopus* to understand human disease and developmental disorders. *Genesis* 55:e22997. 10.1002/dvg.22997 28095616

[B68] Shannon P. Markiel A. Ozier O. Baliga N. S. Wang J. T. Ramage D. (2003). Cytoscape: a software environment for integrated models of biomolecular interaction networks. *Genome Res.* 13 2498–2504. 10.1101/gr.1239303 14597658 PMC403769

[B69] Shi Z. Wang F. Cui Y. Liu Z. Guo X. Zhang Y. (2015). Heritable CRISPR/Cas9-mediated targeted integration in *Xenopus tropicalis*. *FASEB J.* 29 4914–4923. 10.1096/fj.15-273425 26268927

[B70] Sigel E. Minier F. (2005). The *Xenopus* oocyte: system for the study of functional expression and modulation of proteins. *Mol. Nutr. Food. Res.* 49 228–234. 10.1002/mnfr.200400104 15704243

[B71] Simaite D. Kofent J. Gong M. Ruschendorf F. Jia S. Arn P. (2014). Recessive mutations in PCBD1 cause a new type of early-onset diabetes. *Diabetes* 63 3557–3564. 10.2337/db13-1784 24848070

[B72] Simons C. Rash L. D. Crawford J. Ma L. Cristofori-Armstrong B. Miller D. (2015). Mutations in the voltage-gated potassium channel gene KCNH1 cause Temple-Baraitser syndrome and epilepsy. *Nat. Genet.* 47 73–77. 10.1038/ng.3153 25420144

[B73] Smith C. L. Eppig J. T. (2015). Expanding the mammalian phenotype ontology to support automated exchange of high throughput mouse phenotyping data generated by large-scale mouse knockout screens. *J. Biomed. Semantics* 6:11. 10.1186/s13326-015-0009-1 25825651 PMC4378007

[B74] Sobeck A. Stone S. Costanzo V. de Graaf B. Reuter T. de Winter J. (2006). Fanconi anemia proteins are required to prevent accumulation of replication-associated DNA double-strand breaks. *Mol. Cell Biol.* 26 425–437. 10.1128/MCB.26.2.425-437.2006 16382135 PMC1346898

[B75] Staubli A. Capatina N. Fuhrer Y. Munier F. L. Labs S. Schorderet D. F. (2017). Abnormal creatine transport of mutations in monocarboxylate transporter 12 (MCT12) found in patients with age-related cataract can be partially rescued by exogenous chaperone CD147. *Hum. Mol. Genet.* 26 4203–4214. 10.1093/hmg/ddx310 29088427

[B76] Steffensen A. B. Refaat M. M. David J. P. Mujezinovic A. Calloe K. Wojciak J. (2015). High incidence of functional ion-channel abnormalities in a consecutive Long QT cohort with novel missense genetic variants of unknown significance. *Sci. Rep.* 5:10009. 10.1038/srep10009 26066609 PMC4464365

[B77] Stone S. Sobeck A. van Kogelenberg M. de Graaf B. Joenje H. Christian J. (2007). Identification, developmental expression and regulation of the *Xenopus* ortholog of human FANCG/XRCC9. *Genes Cells* 12 841–851. 10.1111/j.1365-2443.2007.01096.x 17584296

[B78] Tandon P. Conlon F. Furlow J. D. Horb M. E. (2017). Expanding the genetic toolkit in *Xenopus*: approaches and opportunities for human disease modeling. *Dev. Biol.* 426 325–335. 10.1016/j.ydbio.2016.04.009 27109192 PMC5074924

[B79] The Gene Ontology Consortium (2017). Expansion of the Gene Ontology knowledgebase and resources. *Nucleic Acids Res.* 45 D331–D338. 10.1093/nar/gkw1108 27899567 PMC5210579

[B80] Ullah G. Demuro A. Parker I. Pearson J. E. (2015). Analyzing and modeling the kinetics of amyloid beta pores associated with Alzheimer’s disease pathology. *PLoS One* 10:e0137357. 10.1371/journal.pone.0137357 26348728 PMC4562663

[B81] Van Nieuwenhuysen T. Naert T. Tran H. T. Van Imschoot G. Geurs S. Sanders E. (2015). TALEN-mediated apc mutation in *Xenopus tropicalis* phenocopies familial adenomatous polyposis. *Oncoscience* 2 555–566. 10.18632/oncoscience.166 26097888 PMC4468341

[B82] Vindas-Smith R. Fiore M. Vasquez M. Cuenca P. Del Valle G. Lagostena L. (2016). Identification and functional characterization of CLCN1 mutations found in nondystrophic myotonia patients. *Hum. Mutat.* 37 74–83. 10.1002/humu.22916 26510092

[B83] Vize P. D. Seufert D. W. Carroll T. J. Wallingford J. B. (1997). Model systems for the study of kidney development: use of the pronephros in the analysis of organ induction and patterning. *Dev. Biol.* 188 189–204. 10.1006/dbio.1997.8629 9268568

[B84] Walentek P. Beyer T. Hagenlocher C. Muller C. Feistel K. Schweickert A. (2015). ATP4a is required for development and function of the *Xenopus* mucociliary epidermis - a potential model to study proton pump inhibitor-associated pneumonia. *Dev. Biol.* 408 292–304. 10.1016/j.ydbio.2015.03.013 25848696 PMC4592800

[B85] Walentek P. Quigley I. K. (2017). What we can learn from a tadpole about ciliopathies and airway diseases: using systems biology in *Xenopus* to study cilia and mucociliary epithelia. *Genesis* 55:e23001. 10.1002/dvg.23001 28095645 PMC5276738

[B86] Wang F. Shi Z. Cui Y. Guo X. Shi Y. B. Chen Y. (2015). Targeted gene disruption in *Xenopus laevis* using CRISPR/Cas9. *Cell Biosci.* 5:15. 10.1186/s13578-015-0006-1 25897376 PMC4403895

[B87] Willis J. DeStephanis D. Patel Y. Gowda V. Yan S. (2012). Study of the DNA damage checkpoint using *Xenopus* egg extracts. *J. Vis. Exp.* 69:e4449. 10.3791/4449 23149695 PMC3514051

[B88] Yaoita Y. Shi Y. B. Brown D. D. (1990). *Xenopus laevis* alpha and beta thyroid hormone receptors. *Proc. Natl. Acad. Sci. U.S.A.* 87 7090–7094. 10.1073/pnas.87.18.7090 2402492 PMC54689

[B89] Yelin R. Schyr R. B. Kot H. Zins S. Frumkin A. Pillemer G. (2005). Ethanol exposure affects gene expression in the embryonic organizer and reduces retinoic acid levels. *Dev. Biol.* 279 193–204. 10.1016/j.ydbio.2004.12.014 15708568

