# Supplementary material for: Xenbase: Facilitating the Use of Xenopus to Model Human Disease
Source: Front Physiol. 2019 Feb 26;10:154. doi: 10.3389/fphys.2019.00154 (PMC6399412; doi:10.3389/fphys.2019.00154)
Supplement: Supplementary file 4 [file Data_Sheet_2.docx]

***Supplementary Material***

**Xenbase: Facilitating the use of *Xenopus* to Model Human Disease**

**Mardi J. Nenni^1^*, Malcolm E. Fisher^1^*, Christina James-Zorn^1^, Troy J. Pells^2^, Virgilio Ponferrada^1^, Stanley Chu^2^, Joshua D. Fortriede^1^, Kevin A. Burns^1^, Ying Wang^2^, Vaneet S. Lotay^2^, Dong Zhou Wang^2^, Erik Segerdell^3^, Praneet Chaturvedi^1^, Kamran Karimi^2^, Peter D. Vize^2^, and Aaron M. Zorn^1^**

*** Correspondence:** Corresponding authors: [aaron.zorn@cchmc.org](mailto:aaron.zorn@cchmc.org) , [mardi.nenni@cchmc.org](mailto:mardi.nenni@cchmc.org) and [malcolm.fisher@cchmc.org](mailto:malcolm.fisher@cchmc.org)

**Supplementary methods**

**Collection of *Xenopus*-human disease articles**

Articles were extracted from PubMed using combinations of the search string "*Xenopus*"[MeSH Terms] OR "*Xenopus*"[All Fields] OR "*Xenopus* laevis"[MeSH Terms] OR ("*Xenopus*"[All Fields] AND "laevis"[All Fields]) OR "*Xenopus* laevis"[All Fields]) AND ("humans"[MeSH Terms] OR "humans"[All Fields] OR "human"[All Fields]) AND ("disease"[MeSH Terms] OR "disease"[All Fields]) AND Model[All Fields]. This query was performed on 17 April 2018 and yielded 226 results.

Additional articles were identified from the Xenbase literature module via Textpresso. A query language search was performed in the Xenbase instance of Textpresso with the following criteria: “set scope=sentence, set mode=Boolean, set sort=score, set field= title,abstract,results,discussion, body, find keyword "human disease" > 0”. This search was performed on 11 April 2018 and yielded 466 results.

**Clustering of DO terms**

An abbreviated simple interaction file (sif) form of the DO (downloaded from <https://github.com/DiseaseOntology/HumanDiseaseOntology/blob/master/src/ontology/doid.obo> on 6/10/2018), consisting solely of ‘Is_a’ relationships, was created using custom perl scripts to parse the doid.obo file. This simplified DO structure was loaded into the Cytoscape (v3.6.1) network visualization software. A table of DO terms with curation values, both for direct curations and for curations including all descendent terms, was used to annotate the network view in Cytoscape. A new network was produced from only those nodes representing DO terms with either direct curations or terms with curated descendant terms and the edges joining the selected nodes, the resulting network is available in Supplementary Data 1. The topology for this network was simplified by removing certain highly connected high-level terms, specifically Disease (DOID:4), Nervous system disease (DOID:863) and Disease of anatomical entity (DOID:7). The new network was then clustered using the MCL clustering algorithm from the clusterMaker2 (v1.2.1) Cytoscape app. The inflation value was set to 3.7, and the resulting network organized with a prefuse force directed layout algorithm followed by manual editing to reduce overlapping edges within clusters. A simplified annotated version of the resulting visualization is in Fig. 4 and the full network is available in Supplementary Data 1.

**Supplementary data**

**Supplementary Data 1.** This is a Cytoscape session file requiring the Cytoscape software to view. This file contains a hierarchically organized view of the annotated DO terms from the analysis described in the accompanying article, a force directed layout of the MCL clustered network detailed above with annotation for subnetworks representing our selected high level DO terms and individual views of each major cluster named with the high level DO terms they encompass. All networks have corresponding data tables with the disease name, descendant attribution count, direct annotation count, and total attribution count for each DO term along with the MCL cluster they belong to.

**Supplementary table legends**

**Supplementary Table S1.** References categorized by disease. This table provides an interactive list of the annotated *Xenopus* human disease articles by disease. The accompanying drop-down menu allows filtering of the references based on the higher-level disease terms used in this paper.

**Supplementary Table S2.** DO attribution count and *Xenopus* experimental system(s) used in human disease modeling as assessed in this review. The DO terms displayed here are represented in a hierarchical structure, where higher-level terms are left justified, and descendant terms are indented below. Some intermediate terms are omitted here for sake of brevity and their descendants are double indented. DO terms are also color coded from highest level (dark blue) to lowest level (white) in each ontology branch shown. Total attributions include direct and indirect annotations (column C). The three experimental assays used in the reviewed articles are indicated by a black asterisk (*): cell free extract (column D), the oocyte (column E) or the whole embryo (column F). The overview of experimental systems used for the umbrella DO term (e.g., nervous system disease) are shown as a white asterisk against the dark blue background.
